# COX-2 as a potential biomarker and therapeutic target in melanoma

**DOI:** 10.20892/j.issn.2095-3941.2019.0339

**Published:** 2020-02-15

**Authors:** Diana Valentina Tudor, Ioana Bâldea, Mihai Lupu, Teodor Kacso, Eniko Kutasi, Andreea Hopârtean, Roland Stretea, Adriana Gabriela Filip

**Affiliations:** ^1^Department of Physiology, University of Medicine and Pharmacy “Iuliu Hațieganu”, Cluj-Napoca 400000, Romania

**Keywords:** Melanoma, inflammation, COX-2, COX-2 inhibitors, celecoxib

## Abstract

With a constantly increasing incidence, cutaneous melanoma has raised the need for a better understanding of its complex microenvironment that may further guide therapeutic options. Melanoma is a model tumor in immuno-oncology. Inflammation represents an important hallmark of cancer capable of inducing and sustaining tumor development. The inflammatory process also orchestrates the adaptative immunosuppression of tumor cells that helps them to evade immune destruction. Besides its role in proliferation, angiogenesis, and apoptosis, cyclooxygenase-2 (COX-2) is a well-known promoter of immune suppression in melanoma. COX-2 inhibitors are closely involved in this condition. This review attempts to answer two controversial questions: is COX-2 a valuable prognostic factor? Among all COX-2 inhibitors, is celecoxib a suitable adjuvant in melanoma therapy?

## Introduction

Metastatic melanoma remains one of the most aggressive and dreaded types of skin cancer, due to its rapid evolution and acquired drug resistance^1^. Because of its continuous increasing incidence, there is an urgent need to find more efficient follow-up and treatment options for melanoma patients^2–4^. Melanoma biology is very complex and only partially understood, therefore in 2012 it was considered a model tumor for immuno-oncology^3^. Clinical practice uses tumor thickness and mitotic rates as reliable prognostic factors. But other more reliable biomarkers to predict the therapeutic response of melanoma are needed^5^.

Hanahan and Weinberg^6^ pointed to inflammation and genomic instability as the two main “hallmarks” through which tumors develop. Chronic inflammation can initiate and sustain tumor development, and in turn, a tumor constantly secretes inflammatory molecules that insures its progression, giving rise to a “boomerang effect”^7,8^. These inflammation-promoting molecules are mostly represented by cytokines, chemokines, prostaglandins (PG) and microRNAs^9–11^.

Cyclooxygenase-2 (COX-2) expression has a pathological significance. High levels of COX-2 isoform have been detected in both murine and human melanoma models^12,13^. COX-2 has been linked to the stimulation of angiogenesis, inhibition of apoptosis, increased cell proliferation, cell invasiveness, immunosuppression, and the production of mutagens. To the best of our knowledge, the first study regarding the role of COX-2 in human malignant melanoma was published in 2001^13–17^. Besides these pro-tumorigenic mechanisms, recent studies indicate that the intense expression of COX-2 has an important role in tumor chemoresistance^18^. Among all prostanoids, PGE_2_ mediated by COX-2 is of great relevance. PGE_2_ plays a critical role in carcinogenesis by interfering with the invasion and progression of malignant melanomas^19^. Therefore, an inflammation blockade in order to prevent or treat cancer becomes a logical approach. A convenient option seems to be the inhibition of COX activity using non-steroidal anti-inflammatory drugs (NSAIDs), either classical or selective [COX-2 inhibitors (COXIBs)].

In this review, we propose an updated overview regarding the role of COX-2 in the progression of melanoma, focused on 1) the relevance of COX-2 as a potential prognostic factor; 2) the potential beneficial role of COX-2 as a therapeutic ­target in melanoma; 3) the use of COX-2 inhibitors, focusing mainly on celecoxib, as adjuvants for current oncological therapies.

## COX-2 pathways in inflammation and cancer

It is well known that the COX enzyme has two isoforms, COX-1 and COX-2. Both COX-1 and COX-2 are able to catalyze prostanoids production: like PGE_2_, PGD_2_, PGF_2_, prostacyclin (PGI_2_), and thromboxane A_2_ (TXA_2_), from arachidonic acid^20^. The central role of COX-2 is to orchestrate chronic inflammation which often leads to the main tumor-promoting signaling pathways, like tumor necrosis factor/tumor necrosis factor-receptor (TNF/TNF-R), epidermal growth factor/epidermal growth factor-receptor (EGF/EGF-R) or S100/receptor for advanced glycation end products (S100/RAGE)^21,22^. The main effects of COX-2 in melanoma are related to PGE_2_ production. Different cytokines, growth factors and endotoxins also induce COX-2 expression. For instance, interleukin-1β (IL-1β) has been reported to be significantly expressed in melanoma. The nuclear factor-κB (NF-κB) transcription factor is involved in IL-1β mediated COX-2 expression with further PGE_2_ release in melanoma cells^23^. COX-2 enzyme and the subsequently produced PGE_2_ contribute to key cellular activities at the tumor milieu, namely proliferation, angiogenesis, immune defense, and apoptosis^19,24,25^. In the case of melanoma, COX-2 increases vascular endothelial growth factor (VEGF) expression in the tumor milieu through the phosphoinositide 3-kinase (PI3K)/protein kinase C (PKC) signaling pathway and thus it promotes tumor-induced angiogenesis. Moreover, *via* activated PI3K, COX-2 induces matrix metalloproteinase (MMP) 14 and 2 that are responsible for the degradation of the extracellular matrix, tumor invasion, and vascular mimicry in melanoma^26,27^. Interestingly, incipient neoplasms behave more like a normal wounded tissue, where COX-2 is expressed by stromal cells^28^. In invasive tumors the regulatory mechanisms are damaged and tumor cells become autonomous, as dysplastic epithelium itself is responsible for COX-2 expression^29^.

## COX-2 and transcription factors in melanoma

COX-2 can be a driver of immune suppression in melanoma, but the exact mechanism is uncertain. One of the most studied pathways in melanoma remains the mitogen-­activated protein kinase (MAPK) pathway, which determines increased levels of the activator protein-1 (AP-1) transcription factor. The MAPK family is composed of extracellular signal-regulated kinase (ERK) 1/2, c-JUN N-terminal kinase (JNK) and p38. During melanoma immunosuppression, *BRAF*^V600E^ oncogenes drive the activation of the MAPK pathway in melanoma cells, with further release of IL-1 α/β. In response to IL-1, tumor associated fibroblasts produce COX-2, programmed death-ligand 1 (PD-L1) and chemokine that maintain T-cell suppression^30^. It seems that COX-2 expression in melanoma tumors positively correlates with PD-L1 expression^31^. Transient levels of reactive oxygen species (ROS) activate MAPK, PI3K/serine/threonine kinase (AKT) and NF-κB proliferative pathways. Sun exposure can lead to ROS-induced JNK and p38 in keratinocytes and human fibroblasts of the exposed skin, with further activation of AP-1 transcription. AP-1 binds to *COX-2* gene promoter and increases *COX-2* gene transcription^32^. AP-1 transcription factor complex (composed of FOS and JUN proteins) has been identified as the main determinant in tumor progression, proliferation, migration, invasion, angiogenesis, and drug resistance^33,34^. Although, AP-1 proteins are primarily considered to be oncogenic, recent studies revealed that JUNB and c-FOS proteins display a tumor-suppressor activity as well^35,36^. Furthermore, the AP-1 family member c-JUN is a key factor involved in melanoma progression, responsible for gene deregulation in MAPK and PI3K pathways^37,38^. Thus, it seems that COX-2 expression and PGE2 production are closely linked to MAPK, as well as the activation of PI3K pathways. Besides, COX-2 and indoleamine 2, 3-dioxygenase 1 (IDO1) are considered “partners in crime” when it comes to the promotion of immune dysfunction and tumor survival in cancers^39,40^. Another path leading to COX-2 production that sustains chronic inflammation and tumor evasion in BRAF^V600E^ positive human melanoma is the Janus kinase-2/signal transducer and activator of transcription 3 (JAK-2/STAT3)^41,42^.

**Figure 1:**
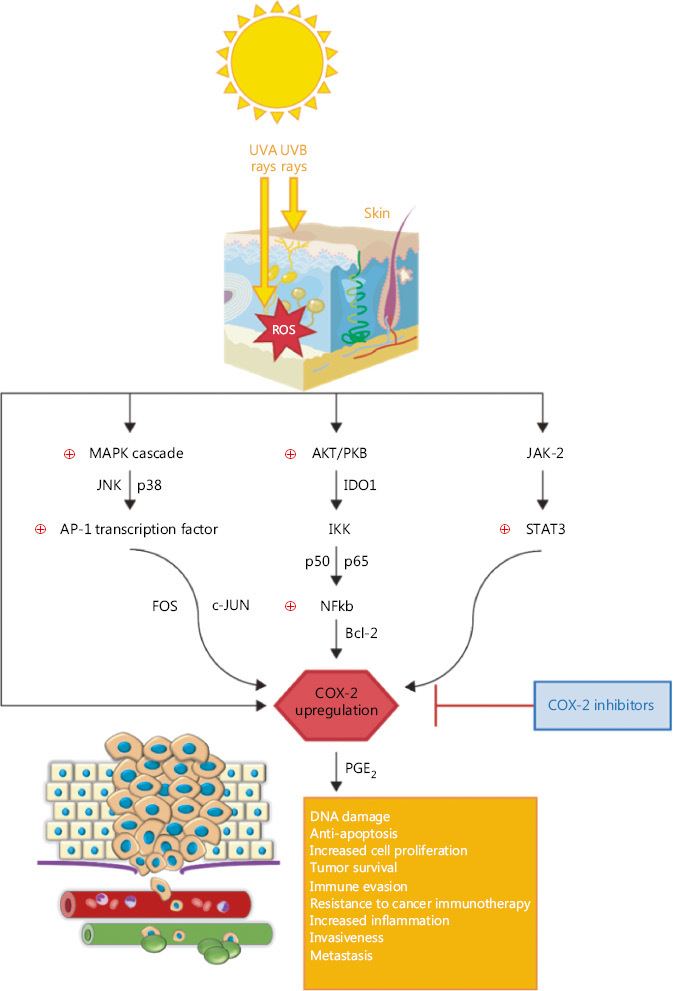
The intricate role of COX-2 in melanoma pathways.

Invasiveness is another important characteristic of melanoma, defined by the loss of adhesion molecules. The cell adhesion molecule E-cadherin facilitates the contact between melanocytes and keratinocytes. The loss of E-cadherin is mediated through the activation or repression of NF-κB ­signaling pathway *via* the β-catenin–p38 axis^43^. Melanoma cells become resistant to apoptosis and further cytotoxic therapies when the NF-κB pathway is activated, using the inhibitor of κB kinase complex (IKK). In the course of melanoma cell proliferation NF-κB complex (p50/p65) is up-regulated after the activation of AKT/PKB, a serine/threonine kinase that is the core component of the PI3K signaling pathway. Furthermore, NF-κB determines the up-regulation of the B-cell lymphoma-2 (Bcl-2) anti-apoptotic protein and COX-2 expression as a result^11,44,45^. The way COX-2 interferes in melanoma pathways is summarized in **Figure 1**. With such an intricate role in melanoma genesis and progression, COX-2 has gained a lot of interest lately and COXIBs became a logical approach to be tested as chemoprevention in melanoma.

Both ultraviolet (UV) A and UVB rays activate the production of high ROS levels in the exposed skin, which can further trigger three important pathways: MAPK cascade (a family of proteins which includes JNK and p38) with further activation of AP-1 transcription factor, composed of FOS and c-JUN proteins; AKT/PKB cascade with modulation of IKK, through the activation of IDO1 and the anti-apoptotic NF-κB (p50 and p65 proteins)-Bcl-2 pathway; JAK-2 and STAT-3 activation. All these pathways are linked to chronic inflammation and promote tumor progression *via* COX-2 upregulation and PGE2 production at the tumor site. The current literature associates COX-2 with DNA damage, resistance to apoptosis and proliferation, tumor survival, immune or immunotherapy resistance, as well as invasiveness and metastasis in melanoma. For this reason, COX-2 inhibitors could be a suitable choice as adjuvants in the therapeutic management of melanoma.

## UV exposure, COX-2 production, and melanogenesis

Repeated UVA and UVB skin damage triggers the production of arachidonic acid in human keratinocytes, with further DNA damage and COX-2 mediated PGE2 production. As a result, this will induce an increased cell replication and decreased apoptosis in melanocytes^46,47^. As a proof, studies performed *in vivo* on genetically COX-2-deficient animals or animals treated with COX-2 inhibitors showed a reduced risk for developing skin tumors when exposed to UV light^48^.

Until 2012, little was known about the effects of COX-2 on pigmentation. Kim et al.^49^ highlighted the link between COX-2 and alpha-melanocyte stimulating hormone (α-MSH) in melanogenesis using short interfering RNA (siRNA). By silencing COX-2 in melanocytes, α-MSH melanin production is decreased, tyrosinase enzyme activity is reduced, as well as tyrosinase-related protein 1 (TRP-1) and TRP-2, glycoprotein (gp)100 and microphthalmia-associated transcription ­factor (MITF) levels. The results were also confirmed in a more recent study where aspirin or celecoxib treatment reduced ­pigmentation in B16, MTG2, and WM3311 cells. Moreover, aspirin and celecoxib did not affect cell viability, but reduced colony formation, cell motility, and melanin production through the suppression of PGE2 and activation of 5′ AMP-activated protein kinase (AMPK) in some of the tested melanoma cell lines^50^.

## COX-2 as a prognostic factor in malignant melanoma

COX-2 certainly plays a more complex role in melanoma than previously thought. Regardless of the type of skin lesions, benign, dysplastic, or malignant, COX-2 expression may differ depending on each case. A retrospective study^51^ showed that all oral and cutaneous melanomas examined by immunohistochemistry were COX-2 positive, while all melanocytic nevi examined were negative. Dysplastic nevi were occasionally COX-2 positive. The authors proposed COX-2 as a marker to distinguish the melanocytic lesions from oral cavity.

At a microscopic level melanomas exhibited an increase in COX-2 expression directly correlated with the anatomical invasion and depth, while benign lesions had their levels decreased with depth. As expected, superficially spreading melanomas register lower levels of COX-2 expression than nodular, ulcerated, or lymph node metastasis melanoma. Moreover, central regions seem to have a stronger COX-2 expression than the periphery, showing an intense chronic inflammatory process right at the core of the tumor^51–53^. Furthermore, another study showed that COX-2 was more markedly expressed in metastatic melanomas than in primary non-metastatic melanomas, thus raising COX-2 as a supplementary and significant negative cell-related prognostic factor^54^.

Results also suggest that COX-2 is associated with the microscopic histopathological parameters of melanomas in general, such as the mitotic index and the Clark level. Soares et al.^54^ observed a strong positive correlation between mitosis index (> 5.5/mm^2^) and high expression of ­COX-2. Microscopic changes also lead to visible alterations at a macroscopic level. COX-2 seems to influence macroscopic characteristics as well, such as: tumor size, Breslow’s depth and ulcerations. Minisini et al.^55^ have shown that COX-2 is highly expressed in melanomas with a high Breslow depth and poor prognosis.

**Figure 2:**
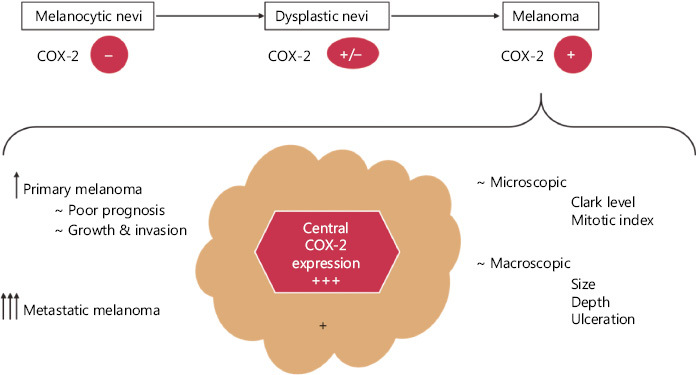
The correlation between COX-2 and clinical features of melanoma.

Through its implications in both macro- and microscopic ­levels, COX-2 could influence the patient’s survival and represent a marker for metastatic development^19^. Meyer et al.^56^ observed a significantly decreased progression-free survival and a tendency to invasion in patients with high levels of COX-2, albeit only in primary metastatic tumors. High metastatic levels of COX-2 were not associated in the same way, which led them to believe that the enzyme may only contribute in the early steps of development and becomes less important as the tumor progresses. They agreed that COX-2 represents a stage-dependent prognostic marker in melanoma, but targeting this enzyme is more a tumor stroma effective approach. A recent study analyzed the levels of COX-2 in 45 lymph nodes with melanoma metastasis and set a threshold for the level of the enzyme expression that affects the patients’ survival. High COX-2 expression (> 10%) reduced progression-free survival by almost 3 years without any correlation to BRAF^V600E^ or NRAS^Q61^ mutations. COX-2 levels above the threshold also allowed for a more aggressive tumor development, leading to a higher probability of metastases. The correlation between COX-2 and the clinical aspects in melanoma was illustrated in **Figure 2**. These clinical findings were further correlated with *in vitro* studies that showed at least 3-fold increased levels of COX-2 in different melanoma cell lines (A375, WM35, WM983A, WM983B, SK-MEL-28, and SK-MEL-5). Moreover, selective COX-2 inhibition using celecoxib managed to reduce cell proliferation and invasiveness in all cell lines tested^57^. A recent study evaluating 77 conjunctival melanomas remarked that tumors with low levels of COX-2 were less likely to end up with poor prognosis, as none of the respective cases had a negative outcome^58^.

COX-2 level differs depending on lesion type: absent/reduced in benign nevi, rarely expressed in dysplastic ones and increased levels in melanoma. Depending on the different melanoma types, primary melanomas express lower levels than the metastatic ones. At the tumor site, COX-2 has an intense expression in the center of the tumor, with reduced COX-2 levels at the periphery. A clear association between COX-2 expression and a tendency to growth and invasion, as well as poor prognosis was remarked in primary melanomas, while in metastatic ones the correlation was not clear. COX-2 was significantly correlated with the microscopic (Clark level and mitotic index) and macroscopic (size, depth, and ulceration) features of the tumor.

Another interesting fact about melanoma patients older than 65 years is the biological behavior of the tumor interacting with an aging immune system: the higher grade of tumor-infiltrating lymphocytes (TILs) at the tumor site, the better the prognosis. COX-2 and PD-L1 were evaluated as potential markers of host immune response and inflammation. This idea was expressed in a retrospective study conducted on elderly patients with early stages of melanoma, where all subjects with COX-2 expression and a lack of PD-L1 expression on TILs registered a worse disease free survival. A possible explanation may be that COX-2 modulates cytokine production on T-cells. Even though COX-2 expression did not correlate with BRAF/NRAS mutation status, it seems that BRAF-mutated melanomas have an increased COX-2 and PD-L1 expression via IL-1 upregulation^58,59^. These results strongly suggest that high levels of COX-2 represent a negative prognostic factor in metastatic melanoma. As it was shown, high COX-2 expression strongly correlates with a deeper Breslow index and a higher rate of lymph node involvement. Therefore, COX-2 is an independent prognostic marker of lower survival outcomes in melanoma^60,61^.

## COX-2 as a target and COX-2 inhibitors in melanoma

In order to overcome the many resistance mechanisms in ­melanoma, drug combinations able to inhibit multiple key pathways in the affected cells could be used as a valid approach for long-term treatment strategy. Some COX-2 inhibitors have been reported to prevent carcinogenesis, therefore selectively blocking this enzyme might be a reasonable choice for chemoprevention or radiosensitization. Later we present several studies that focused on evaluating COX-2 inhibitors’ efficacy used alone or as part of combination therapy in melanoma. COX-2 inhibitors tested in melanoma are summarized in **Table 1**.

The first study to analyze if NSAIDs might have chemopreventive potential against melanoma appeared in 2001 and included only females. Overall, daily intake of aspirin and ibuprofen halved the relative risk of developing malignant melanoma, independent of sun exposure and age. A possible explanation is that NSAIDs promote apoptosis and inhibit cell proliferation, angiogenesis, and mutagenesis^62^.

As previously discussed, one can assume that removing COX-2 from the chain of reactions would hinder or at least diminish the tumorigenic process. Celecoxib used either topically or orally proved a significant chemopreventive activity in UV-induced carcinogenesis in mice due to inhibition of vascular permeability, chronic inflammation, and acute oxidative damage. Unfortunately, neither celecoxib nor indomethacin managed to reduce the UV-induced cell proliferation and edema, while UV-induced PG production was blocked in both cases. These results also conclude that NSAIDs have a reduced therapeutic value when high levels of COX-2 are constitutively expressed by the tumor^63–66^.

COX-2 is involved in melanoma cell proliferation. The inhibition of this enzyme using selective COX-2 inhibitors in BRAF- or NRAS-mutated melanoma may represent a reasonable and clinically feasible option to help inhibit tumor progression and induce tumor cell death^58,67^. Interestingly, the proapoptotic and antiproliferative effects of celecoxib may be independent of COX-2 expression^68^. In this respect, Chuang et al.^69^ demonstrated that COX-2 inhibition is not sufficient to explain the cytotoxic effect of celecoxib in pancreatic carcinoma and glioblastoma cell lines expressing variable levels of COX-2. These affirmations suggest that celecoxib’s action mechanisms are still poorly characterized. Besides, melanoma cells often evade immune control and develop resistance to cancer immunotherapy through COX-2 upregulation and PD-L1 expression. Thus, celecoxib was also able to down-regulate PD-L1^70,71^. Moreover, celecoxib has a unique potency to induce the apoptosis of melanoma cells by generating extensive ROS-production in a dose-dependent manner. This capability could be used to improve the response rate of melanoma patients to chemotherapy^72^.

Muraki et al.^73^ isolated tumor endothelial cells from human melanoma and highlighted that angiogenesis might be the primary target of COX-2 inhibitors. Their results also showed that the PI3K/AKT pathway was blocked using COX-2 inhibitors. Furthermore, Gowda et al.^74^ wanted to target both COX-2 and PI3K by using a synthetized analog of celecoxib, called selenocoxib-1-GSH. The agent led to a synergistic antitumorigenic action by 70%, while it had negligible toxicity through its COX-2 targeting, unlike celecoxib. The final consequence of the inhibition was a significant decrease in cell proliferation (80%) and a substantial increase in apoptosis.

A study conducted by Hennequart et al.^39^ showed how COX-2 shaped the immunosuppressive tumor microenvironment in both melanoma (KUL98-MELA) and non-melanoma cell line tumors. As previously shown in many human tumors carrying MAPK and PI3K mutations, COX-2 and PGE2 expression are closely linked to IDO-1 enzyme transcription in dendritic cells. Moreover, it seems that IDO-1 positive melanoma patients have a poor outcome^75^. As a negative feedback response to the interferon γ (IFNγ) released by T lymphocytes at the tumor site, IDO-1 is activated and degrades tryptophan, further inducing T lymphocytes’ proliferation arrest and ­apoptosis by triggering an adaptive evasion from the immune control^72,76^. As COX-2 and autocrine PGE_2_ induce IDO-1 expression, melanoma cells treated with celecoxib showed a reduction in IDO-1 transcripts levels by 3-fold and no detectable IDO-1 protein. Thus, IDO-1 expression can be prevented by COX-2 or IDO-1 inhibitors, which may represent a reasonable adjuvant choice to current immunotherapy approaches^39,72^.

Tertiary prevention is an area where COX-2 inhibitors could conquer. A recent *in vitro* study conducted on murine B16F10 and human A375 melanoma cell lines proved the synergistic inhibitory effect of protein kinase C ζ (PKCζ) inhibitor J-4 combined with celecoxib. *In vitro*, cell migration mechanisms and cell adhesion were severely impaired, while the mesenchymal-epithelial transition was induced, preventing further metastatic development. Moreover, MMP-2/MMP-9 secretion was significantly reduced under the combined treatment. *In vivo*, the associated therapy proved effective in reducing lung metastatic nodules while almost completely halting melanoma lung metastasis. Moreover, the reduced toxicity of the drug combination paired with its apparent effectiveness and made it a possible candidate for further human studies^77^.

At the tumor microenvironment, high levels of transforming growth factor beta 1 (TGF-β1) activate fibroblasts to express 5-lipoxygenase (LOX), responsible for focal adhesion through collagen cross-linking and cell migration *via* PI3K upregulation^78^. An even more promising agent than celecoxib proved to be Licofelone (a COX-2/5-LOX inhibitor), which improved the therapeutic effect of a liposomal cancer vaccine (tyrosinase-­related protein 2 peptide with α-galactosylceramide) by limiting the inflammatory-induced immunosuppression and ­awakening of the immune defense against melanoma cells, *in vivo*. In addition, they highlighted that Licofelone alone is not able to inhibit melanoma cell growth and ROS production on B16F10luc2 melanoma cells *in vitro*, despite its inhibitory effect on immature myeloid cells generation^79^. Moreover, Licofelone showed a dose-dependent anticancer response by activation of apoptosis and reduction of the immune-resistant tumor cell population and proved to be more efficient than single COX-2 inhibition (celecoxib)^80–82^. Celecoxib has also proven direct effects on mitochondria in metastatic cancer cells. Among other NSAIDs, celecoxib showed particular potency by inducing ROS-dependent apoptosis against melanoma cells. The decrease in cellular respiration and the ROS-production were dose-dependent, making celecoxib a valuable cytotoxic drug for metastatic melanoma^83^.

COX-2 inhibitors were also tested as adjuvants for ­chemotherapy. When celecoxib was used in combination with dacarbazine (DTIC), the chemotherapeutic with immunostimulatory effect in metastatic melanoma, they induced higher apoptosis than each drug used separately^84^. The combination inhibited more pulmonary metastasis, compared to DTIC alone. Although one concern is the potential human toxicity of this combination, celecoxib is able to decrease the therapeutic dosage of DTIC^85^. Recent studies have tried to synthesize new celecoxib analogues with potent cytostatic activity against melanoma cells^86^.

An interesting case report published in 2006 presented the case of a 72-year-old female with stage IV melanoma with multiple skin metastases. Given the lack of response to conventional therapies, she was treated with rofecoxib, a selective COX-2 inhibitor. Interestingly, after 1 year of treatment with rofecoxib all of her cutaneous metastases disappeared, with long-lasting remission. This case also highlighted that progressive melanoma remains sensitive to COX-inhibitors and these agents proved efficient in cutaneous metastases, but not in visceral ones^87^.

**Figure 3:**
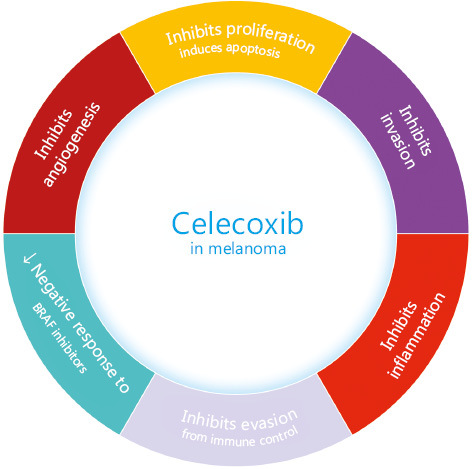
The main effects of celecoxib on melanoma.

Unfortunately, there is a well-known risk of cardiovascular (mainly atherothrombotic) and gastrointestinal side effects among selective COXIBs. As a result, rofecoxib has been withdrawn because of its increased risk of cardiovascular events proved in the APPROVE trial^88^. Celecoxib, on the other hand, has proved a lower risk for cardiovascular events (myocardial infarction and stroke) than other NSAIDs. Celecoxib, the most tested COXIB for its antineoplastic properties, determines AMPK-α and cAMP responsive element binding protein 1 (CREB-1) phosphorylation, promoting vascular endothelial protection. After activating AMPK, TNF-α induced NF-κB p65 expression is inhibited. Celecoxib also prevents the induction of IL-1β mediated expression of IL-6. Moreover, celecoxib promotes the formation of a protective protein heme oxygenase-1 (HO-1) and induces H-ferritin. Thus, it seems that ­celecoxib functions like ibuprofen and naproxen. The main antineoplastic effects of celecoxib are summarized in **Figure 3**. As far as there is a considerable heterogeneity among NSAIDs and COXIBs, each drug should be considered as individual^89^.

Celecoxib has proven to inhibit not only chronic inflammation in melanoma, but also many other tumor hallmarks, like angiogenesis, cell proliferation, invasion, and immune control escape. Moreover, celecoxib reduced the negative response of melanoma cells to BRAF inhibitors.

## The importance of COX-2 for immunotherapy in melanoma

One of the most important mechanisms of tumor immune escape in melanoma is represented by the PD-1 and PD-L1/PD-L2 interaction. This further aids T-cell tolerance and contributes to uncontrolled tumor growth. Anti-PD-1 checkpoint inhibitors have revolutionized the treatment of metastatic melanoma, but there are still large numbers of patients who fail to respond because of PD-L1 upregulation. As previously mentioned, the COX-2/ PGE_2_ pathway positively modulates PD-L1 expression *via* PI3K/AKT, NF-κB and STAT3 activation. Thus, COX-2 is also a resistance ­factor against antigen-specific T-cell cytotoxicity^31^. Moreover, it seems that the overexpression of Yin Yang 1 (YY1) transcription factor has an intricate role in COX-2/PGE_2_/PD-L1 expression^90^. Important evidence suggests that available COX-2 inhibitors act synergistically with anti-PD-1 therapy in breast cancer and murine melanoma models. In order to further characterize this synergism, Ferreira et al.^91^ conducted a recent *in vivo* study on C57BL6/j mice that aimed the comparison between anti-PD-1 and ibuprofen + anti-PD-1 therapy. The results showed a significant difference in tumor volume and survival curves between the 2 groups, completing the existing evidence that NSAIDs may serve as a promising, convenient and safe option of enhancing the response to anti-PD-1 therapies.

## Clinical trials that employed COX-2 inhibitors in melanoma

Immune response stimulation against tumor cells is still an intense studied strategy to fight cancer. The effects of COXIBs as adjuvants in different cancers including melanoma represent an area of interest for clinical studies as well. Two clinical trials conducted in the US tested whether developing a vaccine based on the antigens found on the surface of cancer cells would produce an immune response strong enough to destroy tumor cells. Patients with pleural malignancies, epithelial malignancies, sarcoma, and melanoma were included in the study, and were given cyclophosphamide and celecoxib 7 days prior to vaccine administration. The conclusion is yet to be reached^92,93^. In another trial, patients with metastatic melanoma were treated with autologous dendritic cells (DCs) along with human telomerase reverse transcriptase (hTERT) and tumor lysate [HLA-A2 (−)] or p53-derived peptides [HLA-A2 (+)]. DCs are capable of generating a specific immune response *in vivo* and therefore they were used as a vaccine basis. Meanwhile these patients were treated with cyclophosphamide, IL-2 and celecoxib. The overall survival rate increased for the patients who were part of this trial: 18.4 months for patients with a stable disease and 5 months for patients with progressive disease. The mean overall survival time turned out to be 9 months^94^. Another interesting phase II trial that started in 2018 intends to evaluate the antiproliferative potential of aspirin associated to PD-1 (pembrolizumab) and CTLA4 (ipilimumab) inhibitors in patients with unresectable stage III or stage IV melanoma^95^. The results may help clarify the NSAIDs effectiveness in melanoma.

A report written by Wilson ^96^ drew attention to a series of 27 cases of incurable metastatic melanoma that obtained spontaneous regression after celecoxib treatment. Although COX-2 inhibition in melanoma is real, celecoxib’s pro-apoptotic effect seems to be rather independent of COX-2.

## Conclusions

Melanoma develops drug resistance as it progresses. In order to circumvent this major problem, drug combinations able to inhibit multiple key pathways in the affected cells could be used as a valid approach for long-term treatment. COX-2 has an important role in melanoma progression and chemoresistance. Moreover, COX-2 is definitely a negative prognostic marker. Undergoing studies try to identify specific COX-2 inhibitors that could be used against melanoma cells. COXIBs were reported to prevent carcinogenesis and were used as chemotherapeutic agents with encouraging results in melanoma. Selectively blocking COX-2 may be an effective chemopreventive/adjuvant strategy, a hypothesis already tested in clinical trials on melanoma patients. Celecoxib proved to be a suitable adjuvant in melanoma preclinical studies. Despite its incompletely understood antitumor mechanisms, celecoxib has gained a lot of attention given its potent antitumor capabilities. Further clinical evaluation of long-term drug exposure to celecoxib as an adjuvant is necessary.

## Figures and Tables

**Table 1:** Main COX-2 inhibitors tested in melanoma and their effect as possible therapeutic adjuvants

Study type	Cells type	Mutation	COX-2 inhibitor	Effects
*In vitro*^50^	MTG2	NRAS^Q61R^	Aspirin or celecoxib	Inhibits colony formation and migration
	MTG4	BRAF^V600E^		Inhibits melanin synthesis
	MTG5	TP53^R213X^ and PDGFR-AD^846N^		
	A375	BRAF^V600E^		
	B16-F10			
	YUSAC-2 (YU2)			
*In vivo*				Delayed tumor development
				Inhibits proliferation of sensitive tumors
				Suppresses PGE_2_ and activates AMPK
*In vitro*^84^	B16-F10		Celecoxib	Dose dependent ROS-induced apoptosis
*In vitro*^39^	KUL98-MELA	BRAF^V600E^	Celecoxib	Reduced IDO-1 expression, may improve immunotherapy response
*In vitro*^31^	A375	BRAF^V600E^	Celecoxib	PD-L1 and COX-2 down-regulation
	SK-MEL-2	NRAS^Q61R^		Inhibits tumor growth, prevents cell proliferation, induces cell death
*In vitro*^58^	SK-MEL-5	BRAF^V600E^	Celecoxib	Reduced cellular proliferation and invasiveness
		NRAS^Q61^		COX-2 overexpression is a negative prognostic factor
*In vitro*	WM35	BRAF^V600E^	Selenocoxib-1-GSH (analog of celecoxib)	Inhibits cell proliferation
	WM115			Induces G0-G1 cell cycle arrest and apoptosis
	WM27.1			
	A375M			
	1205Lu			
*In vivo*^75^	UACC903			Inhibits PI3K/AKT signaling
	1205 Lu and UACC903			
Case report^88^	Nodular melanoma		Rofecoxib	Complete and long-lasting regression of skin metastasis
